# Influence of active and passive derotation techniques of OMT Kalternborn-Evjenth manual therapy on trunk morphology of adolescents with idiopathic scoliosis – pilot studies

**DOI:** 10.1186/1748-7161-8-S1-O22

**Published:** 2013-06-03

**Authors:** J Durmala, B Wnuk, I Blicharska, K Wadolowski, S Dybula, J Dzierzega

**Affiliations:** 1 Department of Rehabilitation, Medical University of Silesia, Katowice, Poland

## Aim

The aim of this study was to assess the direct impact of different derotation techniques (OMT Kalternborn-Evjenth) on the trunk morphology.

## Methods

This is a prospective, randomized, and double blind study. 17 in-patient adolescents with idiopathic scoliosis treated with DoboMed (DM) were studied for trunk morphology (surface topography – ATR, Hump Sum, absolute rotation, total rotation, POTSI and clinical examination – ATR by scoliometer, kyphosis and lordosis by plurimeter). Measurements were taken in the morning, before (first study), and after (second study) mobilizations, lasting 10 minutes. They were used as a preparatory phase for a series of DoboMed exercises. On the first day, no mobilization was performed – manual therapy was simulated (placebo group). In the second and third day, (for a random order of individual patients – 8 group vs. 9 group) we used passive derotation mobilization techniques (“passive” group) or active (“active” group) only in the area of lumbar curvature. Derotation mobilization techniques were implemented, in accordance with the concept of OMT Kalternborn-Evjenth, in a sitting position. Clinical examination of the lumbar spine was performed in a standing position. Surface topography examination was performed in a sitting position. Statistical analysis was performed using nonparametric tests.

## Results

After performing mobilization techniques, there was a statistically significant reduction in the parameters values (absolute values) (scoliometer ATR, surface topography – ATR, Hump Sum, absolute rotation, total rotation) only in the active and passive group (first study vs second study). Improvement (as a percentage of patients with improvement = relative values) was observed in all measured parameters between the group of mobilization (passive and active) and the placebo group. Only in the POTSI case, improvement was registered (insignificant) only in the active group; and in the case of the lordosis value, there were no statistically significant changes. Better percentage improvement was observed (not statistically significant) for most parameters in the active vs. passive group.

## Conclusions

Derotational mobilization techniques may be useful in the treatment of scoliosis therapy (used as a preparatory phase before the relevant active exercises of 3D correction). The use of derotational active mobilization techniques is slightly more effective than passive. Topic requires further investigation.
